# Case report: Area of focus of management of severe pityriasis rubra pilaris by dose optimization of adalimumab biosimilar in combination with acitretin and montelukast

**DOI:** 10.3389/fmed.2023.1295777

**Published:** 2023-11-30

**Authors:** Kristine Heidemeyer, S. Morteza Seyed Jafari, Lena Farnina, Simon Bossart, Laurence Feldmeyer, Nikhil Yawalkar

**Affiliations:** Department of Dermatology, Inselspital, Bern University Hospital, University of Bern, Bern, Switzerland

**Keywords:** pityriasis rubra pilaris, adalimumab, biosimilar, dose-optimization, phospholipase A2, montelukast

## Abstract

Pityriasis rubra pilaris (PRP) is a rare inflammatory skin disorder characterized by hyperkeratotic follicular papules, orange-red scaling plaques with islands of sparing and palmoplantar keratoderma. While spontaneous resolution occurs in some cases, treatment can be challenging for others. The use of biologics in PRP management has gained attention in recent studies, although their high costs and potential side effects present limitations. We present a case of a 71-year-old patient with treatment-resistant PRP who showed significant improvement through optimized adalimumab treatment. Considering the emerging role of phospholipase A2 in PRP pathogenesis, montelukast was added, further enhancing the therapeutic response. By maintaining montelukast and prolonging the adalimumab interval to 3 and 4 weeks, effective dose optimization was achieved without PRP relapse. This case report highlights the potential for adalimumab dose optimization by shortening the initial treatment interval for increased effectiveness and lengthening the interval during the maintenance phase to conserve medication doses. Montelukast appears to assist in sustaining clinical outcomes during interval prolongation, necessitating further investigation through additional studies.

## Introduction

Pityriasis rubra pilaris (PRP) is a rare inflammatory disease characterized by hyperkeratotic follicular papules and orange-red scaling plaques with islands of sparing on the entire body, as well as palmoplantar keratoderma. It also involves the scalp, face, and nails (1–3). Six clinical subtypes of PRP have been described in adults and children, with type I – the classic adult type – representing the most frequent variant (4). While spontaneous resolution within three years is possible, treating persistent cases can be challenging (2). Systemic retinoids are recommended as first-line therapy, with conventional immunosuppressants being the second-line option in treatment-resistant cases (1, 3). Biologics are often proposed as a third-line treatment. Case reports and real-life studies are important to improve the management of chronic skin diseases, reporting data which may improve treatment decision-making process in daily dermatologic practice (5, 6). Recently, several studies have described the role of phospholipase A2 in the pathogenesis of PRP (7, 8).

This article presents a case of treatment-resistant PRP that exhibited a positive response to an optimized dose of biosimilar adalimumab in combination with acitretin and the leukotriene antagonist montelukast.

## Case description

**Figure 1:**
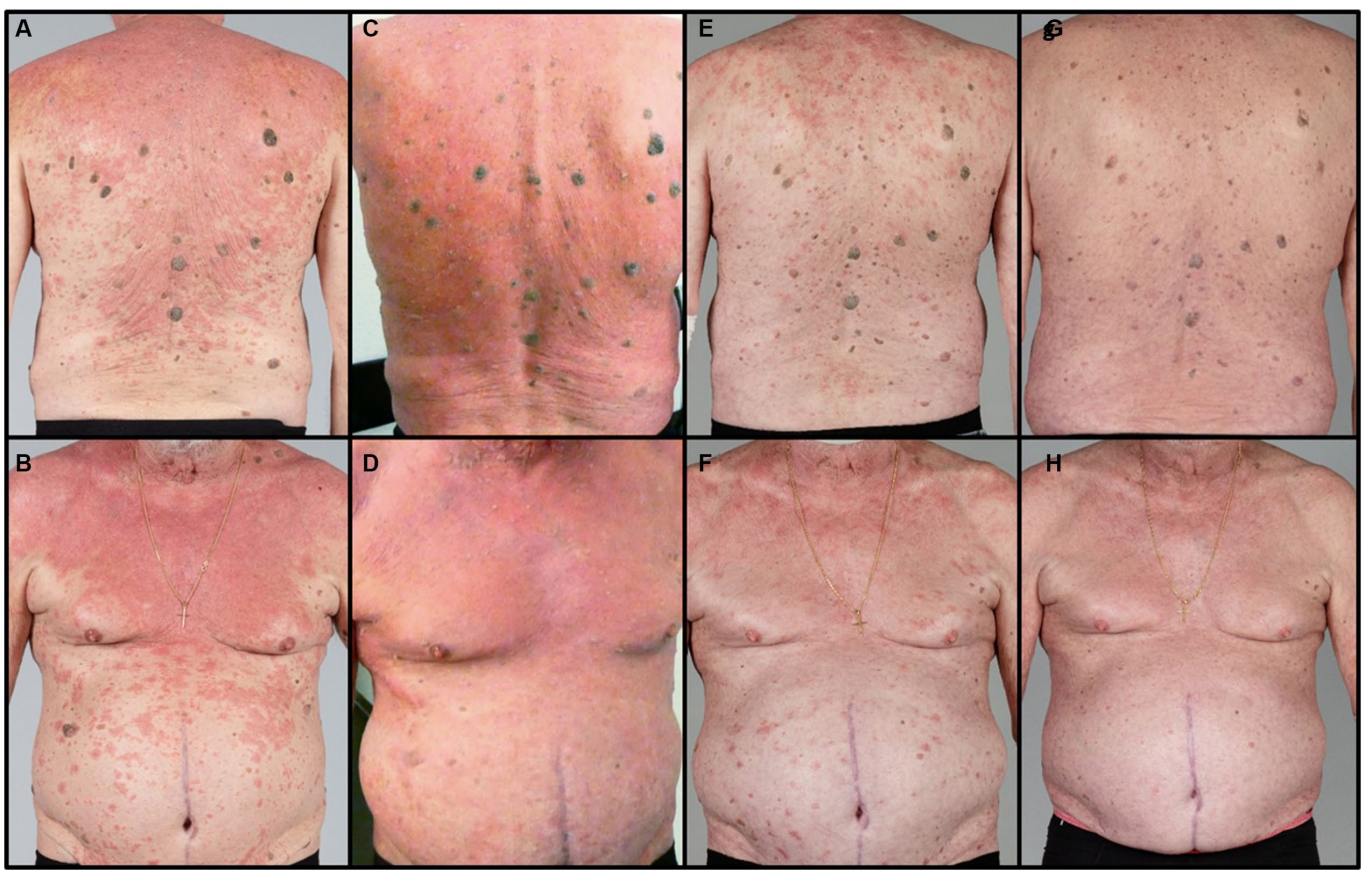
Clinical course: **(A,B)** under treatment with acitretin 25 mg/d and topical steroids, **(C,D)** 4 weeks after initiation of adalimumab biosimilar, **(E,F)** 2 months of increased interval 1/week and addition of montelukast 10 mg/d, **(G,H)** 4 months after dose optimization of adalimumab to 2 weeks and 3 weeks.

**Figure 2:**
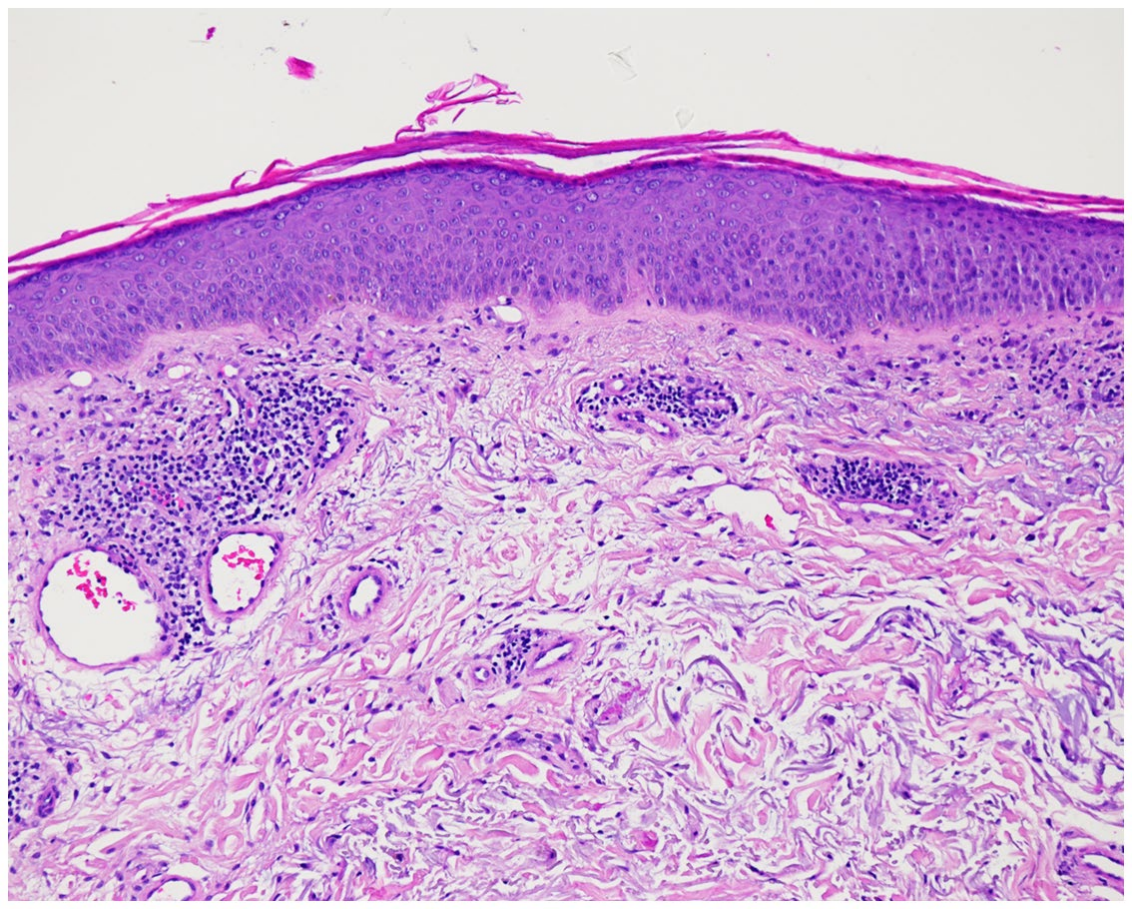
Histopathologic findings showing acanthosis, alternated areas of parakeratosis and orthokeratosis and a dermal mainly perivascular lymphoplasmatic infiltrate (original magnification × 100; hematoxylin and eosin [H&E]).

A 71-year-old patient presented at the Department of Dermatology with generalized, itchy red-orange plaques exhibiting islands of unaffected skin. This condition also involved the face and had developed over the past 8 months (Figures 1A,B). Incidental findings included multiple seborrheic keratosis on the back and chest. The patients also had further comorbidities including pulmonary alveolar proteinosis, a history of sigmoid diverticulitis, metabolic-toxic liver damage, arterial hypertension, peripheral arterial occlusive disease, and chronic obstructive pulmonary disease (COPD). Histopathological examination revealed acanthosis, alternated areas of parakeratosis and orthokeratosis and a dermal mainly perivascular lymphoplasmatic infiltrate (Figure 2). No classical histopathological features of psoriasis as enlarged epidermal ridges and isolated neutrophils or in microabscesses of the epidermis were observed. Based on the typical clinical presentation and compatible histopathological examination, a diagnosis of PRP was established. Based on the histopathological findings the initially considered differential diagnosis of psoriasis and chronic eczema could be excluded. Initial treatment with topical steroids (clobetasol propionate ointment) resulted in only minor improvement. Subsequently, acitretin 25 mg/d was initiated. However, after one month of treatment, due to a lack of improvement and severe itching, adalimumab biosimilar (Hyrimoz®) 40 mg s.c. every 2 weeks was added to the regimen. As skin lesions worsened a month later, we decided to shorten the interval for adalimumab administration to once weekly (Figures 1C,D).

After an additional month, the itch decreased slightly, and skin lesions exhibited only partial improvement. Consequently, montelukast 10 mg/d was introduced as an additional treatment. Within 4 weeks, both the skin lesions and pruritus showed significant improvement, enabling a reduction in adalimumab dosage to 40 mg every 2 weeks (Figures 1E,F). Two months later, only slight postinflammatory erythema was observable on the trunk, with no active PRP manifestations. This progress allowed for a further reduction in adalimumab dosage to 40 mg every 3 weeks while maintaining acitretin and montelukast. Notably, there were no disease flares during the subsequent 4 months of follow-up (Figures 1G,H).

## Discussion

Treating PRP poses ongoing challenges. Due to its rarity, the potential for spontaneous remission and the absence of specific scoring tools, there is a dearth of clinical trials and comparative studies addressing treatment options (2). At the same time, numerous case reports and case series have highlighted the positive impact of TNFα inhibitors, as well as other biologics such as IL-12/23 inhibitors, IL-17 inhibitors, and IL-23 inhibitors, in both adults and children with PRP, either as standalone or combined therapies (1–3, 9–13), Pathogenically, the IL-23/IL-7 axis appears to play a significant role (1, 14). Gain-of-function mutations in the CARD14 gene, notably associated with familial PRP in humans, have led to heightened IL-17 and IL-23 inflammation in mice (1, 14). Reviews that compare the effectiveness of systemic therapies, particularly biologics alone or in conjunction with conventional treatments, have demonstrated complete or excellent responses as follows (2, 3): infliximab 80 and 57.1%, etanercept 81.8 and 53.3%, adalimumab 77.8 and 46.4%, and ustekinumab 92.3 and 62.5% (2, 3). Adalimumab treatment displayed the quickest onset of action (within 3.5 weeks) and exhibited greater efficacy in males (2). TNFα inhibitors yielded better results in TNFα-non-experienced patients (2). In comparison, a noteworthy 42% demonstrated an excellent response to retinoids (2). Clinical trials assessing the impact of IL-17 inhibitors revealed that secukinumab led to a 55% achievement of PASI75 by week 28, while ixekizumab produced a 42% PASI75 response by week 24 (7, 14). However, it’s important to note that while clinical trials exist specifically for IL-17 inhibitors, reports of other biologics’ efficacy rely mainly on case reports and series, necessitating cautious comparison of these results.

Our patient experienced a PRP exacerbation despite receiving acitretin 25 mg/d and adalimumab biosimilar at the conventional dose of 40 mg biweekly. Previous research has demonstrated the potential for optimizing adalimumab dosage through interval adjustments in psoriasis patients, with dose escalation to a weekly regimen leading to notable enhancements in skin lesions (15). The leukotriene receptor antagonist montelukast was introduced to stabilize and further improve the condition. Phospholipase A2 (PLA2) is implicated in multiple inflammatory processes and generates significant lipid mediators like prostaglandins and leukotrienes. Recent studies have highlighted the role of PLA2 in PRP pathogenesis by affecting keratinocytes, immune responses, and epidermal thickness (7, 8). In a clinical trial evaluating secukinumab efficacy in PRP, elevated PLA2 levels were observed in non-responding individuals before treatment (7, 8). While it remains unclear whether leukotrienes induced by PLA2 are responsible for the adverse impact on PRP, our patient experienced improved skin lesions after initiating the leukotriene antagonist montelukast, and these results were sustained during adalimumab dosage reduction. It is worth noting that a limitation of this report is the potential for spontaneous resolution in this patient. Nonetheless, the evident temporal correlation between the treatment initiation and symptom improvement renders this possibility less likely.

## Conclusion

This case report reinforces the significance of utilizing biologics in combination with acitretine and as reported here for the first time with a leukotriene antagonist in PRP treatment, underlining their notable efficacy and safety in managing this frequently treatment-resistant condition. However, the considerable cost associated with biologics likely presents a key deterrent against their adoption as second- or potentially even first-line treatments for PRP. The possibility of dose spacing with biologics may help alleviate this concern. Further research is warranted to examine the impact of leukotriene antagonists and PLA2 inhibitors in PRP treatment.

## Data availability statement

The original contributions presented in the study are included in the article/supplementary material, further inquiries can be directed to the corresponding author.

## Ethics statement

As it is a case report no proof of ethics committee is needed. The studies were conducted in accordance with the local legislation and institutional requirements. The participants provided their written informed consent to participate in this study. Written informed consent was obtained from the individual(s) for the publication of any potentially identifiable images or data included in this article. Written informed consent was obtained from the participant for the publication of this case report.

## Author contributions

KH: Conceptualization, Writing – original draft. SS: Data curation, Writing – review & editing. LeF: Data curation, Writing – review & editing. SB: Data curation, Writing – review & editing. LaF: Data curation, Writing – review & editing. NY: Conceptualization, Writing – review & editing.

## References

[1] Guenther J Novack D Kamath S Worswick S. Treatment options for juvenile Pityriasis Rubra pilaris. Paediatr Drugs. (2023) 25:151–64. doi: 10.1007/s40272-022-00549-4, PMID: 36529810

[2] Kromer C Sabat R Celis D Mossner R. Systemic therapies of pityriasis rubra pilaris: a systematic review. J Dtsch Dermatol Ges. (2019) 17:243–59. doi: 10.1111/ddg.13718 30520557

[3] Naidoo A Sibbald C Fleming PJ Piguet V. Use of biologics in Pityriasis Rubra pilaris refractory to first-line systemic therapy: a systematic review [formula: see text]. J Cutan Med Surg. (2020) 24:73–8. doi: 10.1177/1203475419887731 31691587

[4] Ringin SA Daniel BS. Treatment modalities for pityriasis rubra pilaris subtypes: a review. J Dermatolog Treat. (2022) 33:587–8. doi: 10.1080/09546634.2020.1729954, PMID: 32073340

[5] Megna M Camela E Battista T Genco L Martora F Noto M. Efficacy and safety of biologics and small molecules for psoriasis in pediatric and geriatric populations. Part I: focus on pediatric patients. Expert Opin Drug Saf. (2023) 22:25–41. doi: 10.1080/14740338.2023.2173170, PMID: 36718762

[6] Ruggiero A Potestio L Cacciapuoti S Gallo L Battista T Camela E. Tildrakizumab for the treatment of moderate to severe psoriasis: results from a single center preliminary real-life study. Dermatol Ther. (2022) 35:e15941. doi: 10.1111/dth.15941 36239544

[7] Boudreaux BW Pincelli TP Bhullar PK Patel MH Brumfiel CM Li X. Secukinumab for the treatment of adult-onset pityriasis rubra pilaris: a single-arm clinical trial with transcriptomic analysis. Br J Dermatol. (2022) 187:650–8. doi: 10.1111/bjd.21708 35701384

[8] Shao S Chen J Swindell WR Tsoi LC Xing X Ma F. Phospholipase A2 enzymes represent a shared pathogenic pathway in psoriasis and pityriasis rubra pilaris. JCI Insight. (2021) 6:1911. doi: 10.1172/jci.insight.151911 PMC8564909 34491907

[9] Song EJ Al-Saedy MA Bouche N. Refractory pityriasis rubra pilaris treated with upadacitinib. JAAD Case Rep. (2023) 35:112–4. doi: 10.1016/j.jdcr.2023.03.004, PMID: 37168026 PMC10165156

[10] Ying Y Yu-Hua L Xiao-Yan W Su-Chun H. A case of pityriasis rubra pilaris treated with tofacitinib after failure with acitretin and ixekizumab. Australas J Dermatol. (2023) 64:445–7. doi: 10.1111/ajd.14076, PMID: 37200390

[11] Licata G Gambardella A Calabrese G Pagliuca F Alfano R Argenziano G. Refractory type I pityriasis rubra pilaris treated with tildrakizumab. Clin Exp Dermatol. (2021) 46:1594–5. doi: 10.1111/ced.14790, PMID: 34101231

[12] Feldmeyer L Mylonas A Demaria O Mennella A Yawalkar N Laffitte E. Interleukin 23-helper T cell 17 Axis as a treatment target for Pityriasis Rubra pilaris. JAMA Dermatol. (2017) 153:304–8. doi: 10.1001/jamadermatol.2016.5384 28122069

[13] Fouarge AL Cuvelier M Roquet-Gravy C de Montjoye L Baeck M. Successful treatment of pityriasis rubra pilaris with risankizumab, a IL-23/p19 antagonist. J Eur Acad Dermatol Venereol. (2023) 37:e106–9. doi: 10.1111/jdv.18516, PMID: 35972816

[14] Haynes D Strunck JL Topham CA Ortega-Loayza AG Kent G Cassidy PB. Evaluation of Ixekizumab treatment for patients with Pityriasis Rubra pilaris: a single-arm trial. JAMA Dermatol. (2020) 156:668–75. doi: 10.1001/jamadermatol.2020.0932, PMID: 32293641 PMC7160757

[15] Benzaquen M Munshi M Bossart S Feldmeyer L Emelianov V Yawalkar N. Long-term dose optimization of adalimumab via dose spacing in patients with psoriasis. Bioengineering (Basel). (2022) 9:387. doi: 10.3390/bioengineering9080387 36004912 PMC9405054

